# Ceramide Synthase 6 Deficiency Enhances Inflammation in the DSS model of Colitis

**DOI:** 10.1038/s41598-018-20102-z

**Published:** 2018-01-26

**Authors:** Kristi Helke, Peggi Angel, Ping Lu, Elizabeth Garrett-Mayer, Besim Ogretmen, Richard Drake, Christina Voelkel-Johnson

**Affiliations:** 1 Departments of Comparative Medicine, Medical University of South Carolina, Charleston, SC USA; 2 Pharmacology, Medical University of South Carolina, Charleston, SC USA; 3 Microbiology & Immunology, Medical University of South Carolina, Charleston, SC USA; 4 Public Health Sciences, Medical University of South Carolina, Charleston, SC USA; 5 Biochemistry & Molecular Biology, Medical University of South Carolina, Charleston, SC USA

## Abstract

Colitis, an inflammatory disease of the digestive tract, is increasing in incidence and prevalence. Intestinal inflammation can occur as a consequence of dysfunctions in sphingolipid metabolism. In this study we used ceramide synthase 6 (CerS6) deficient mice, which have a reduced ability to generate long chain C_16_-ceramide, to investigate the role of this enzyme in dextran sodium salt (DSS)-induced colitis. While CerS6-deficient mice are protected from T cell mediated colitis, in the T cell independent DSS model lack of CerS6 resulted in a more rapid onset of disease symptoms. CerS6-deficient mice maintained low levels of C_16_-ceramide after DSS treatment, but the inflammatory lipid sphingosine-1-phosphate was significantly increased in colon tissue. In the absence of CerS6, DSS induced more severe pathology in the colon including enhanced neutrophil infiltration. *In vivo* analysis of myeloperoxidase activity, an enzyme released from neutrophils, was approximately 2.5-fold higher in CerS6-deficient mice compared to wild type. Differences in intestinal permeability did not account for the increase in neutrophils. Our study suggests that lack of CerS6 expression differentially impacts the development of colitis, depending on the model used.

## Introduction

Inflammatory bowel diseases are emerging as a global problem with increased incidence and prevalence in numerous countries^1^. Sphingolipid metabolism has been shown to play a role in colitis and the bioactive lipid sphingosine-1-phosphate (S1P) is emerging as a novel therapeutic target^2–4^. S1P is generated by two sphingosine kinases (SK1, SK2) and SK1 has specifically been associated with colitis in the DSS model as SK1-deficient mice are protected and the novel SK1 inhibitor LCL-351 reduces immune responses^5,6^. S1P is generated from ceramide, which is central to sphingolipid metabolism and varies in acyl chain length ranging from 14–30 (or more) carbons^7^. This variety in acyl chain length derives from the activity of six ceramide synthases (CerS) with different substrate preferences^8^. Through the combined activity of multiple CerS, cells are endowed with a specific ceramide profile presumably required for maintenance of sphingolipid homeostasis in any given tissue. Alterations in ceramide profiles have been observed in multiple diseases, suggesting that dysregulation of CerS may contribute to onset or progression of pathologies^9,10^.

Ceramide synthase 6 (CerS6) is an enzyme that preferentially utilizes 16-carbon fatty acids to generate C_16_-ceramide. CerS6 mRNA is highly expressed in the intestine and immune system^8^. CerS6-deficient mice are viable and except for some behavioral abnormalities, do not display any obvious defects under normal conditions^11^. Different pathologies emerge in disease models however. Lack of CerS6 increases aggressiveness of experimental autoimmune encephalomyelitis (EAE), a model of multiple sclerosis, diminishes macrophage infiltration and pro-inflammatory gene expression in the diet-induced obesity model, and reduces graft-vs-host disease^12–14^. These opposing consequences of CerS6-deficiency across models underscore the complexity of sphingolipid signaling. Recently, adoptive transfer of CerS6-deficient naïve CD4^+^ T cells was shown to protect mice from colitis^12,15^. In this study, we further elucidate the role of CerS6 in DSS-induced colitis, which is independent of T lymphocytes.

## Results

### Wild type and CerS6-deficient mice develop DSS-induced colitis

**Figure 1:**
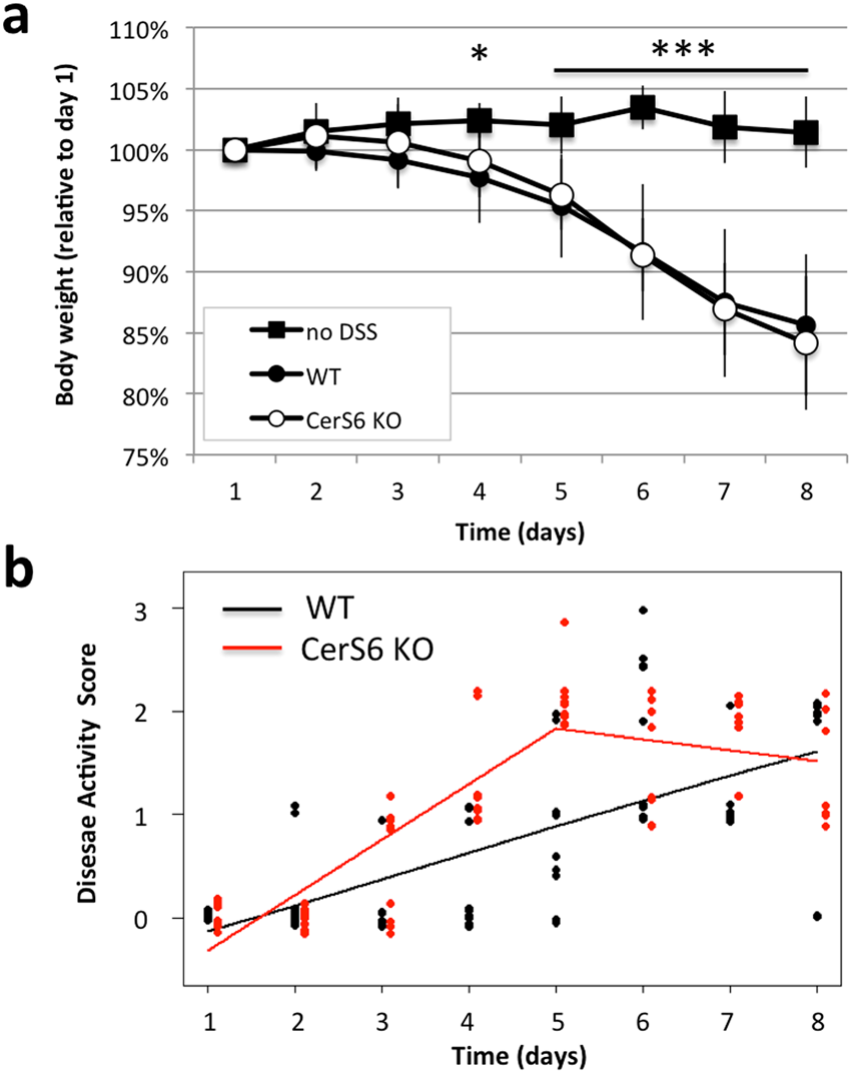
DSS-induced colitis in wild type and CerS6-deficient mice. (**a**) Body weight and (**b**) disease activity score based on analysis of feces. Data shown are from 3 independently performed experiments, each with at least n = 4 per group. The untreated group in (**a**) included both wild type and CerS6-deficient animals. In (**b**), the difference in slopes prior to day 5 was p = 0.0001 and p = 0.0007 between days 5–8.

Colitis was induced by adding 3% DSS for 5 days to the drinking water, followed by 3 days of regular water. Body weight and appearance of feces were monitored daily throughout the 8 day experiment. Beginning on day 4, both wild type and CerS6 deficient mice receiving water supplemented with DSS experienced significant weight loss (Fig. 1a). There was no difference in weight loss between wild type and CerS6 deficient mice or between genders. In addition to weight, we monitored the development of diarrhea and assigned a disease activity score. Data analysis using the student t test indicated that CerS6-deficient mice had significantly higher disease activity on days 4 and 5 compared to wild type mice (p = 0.0007 for day 4 and p = 0.0001 for day 5). Further statistical analysis demonstrated that the slopes describing disease activity trends differed between groups. In wild type mice, disease activity score increased over time in a linear manner with slopes of 0.25 and 0.24 for days 1 to 5 and 5 to 8, respectively. In CerS6-deficient mice disease activity index increased more rapidly over the first 5 days (slope 0.54) and then remained relatively constant (slope -0.11) (Fig. 1b**)**. Thus DSS-induced colitis resulted in similar weight loss and disease activity index by day 8 but progressed with different kinetics in wild type and CerS6-deficient mice.

### Sphingosine-1-phosphate is increased in colons of CerS6-deficient mice

**Figure 2:**
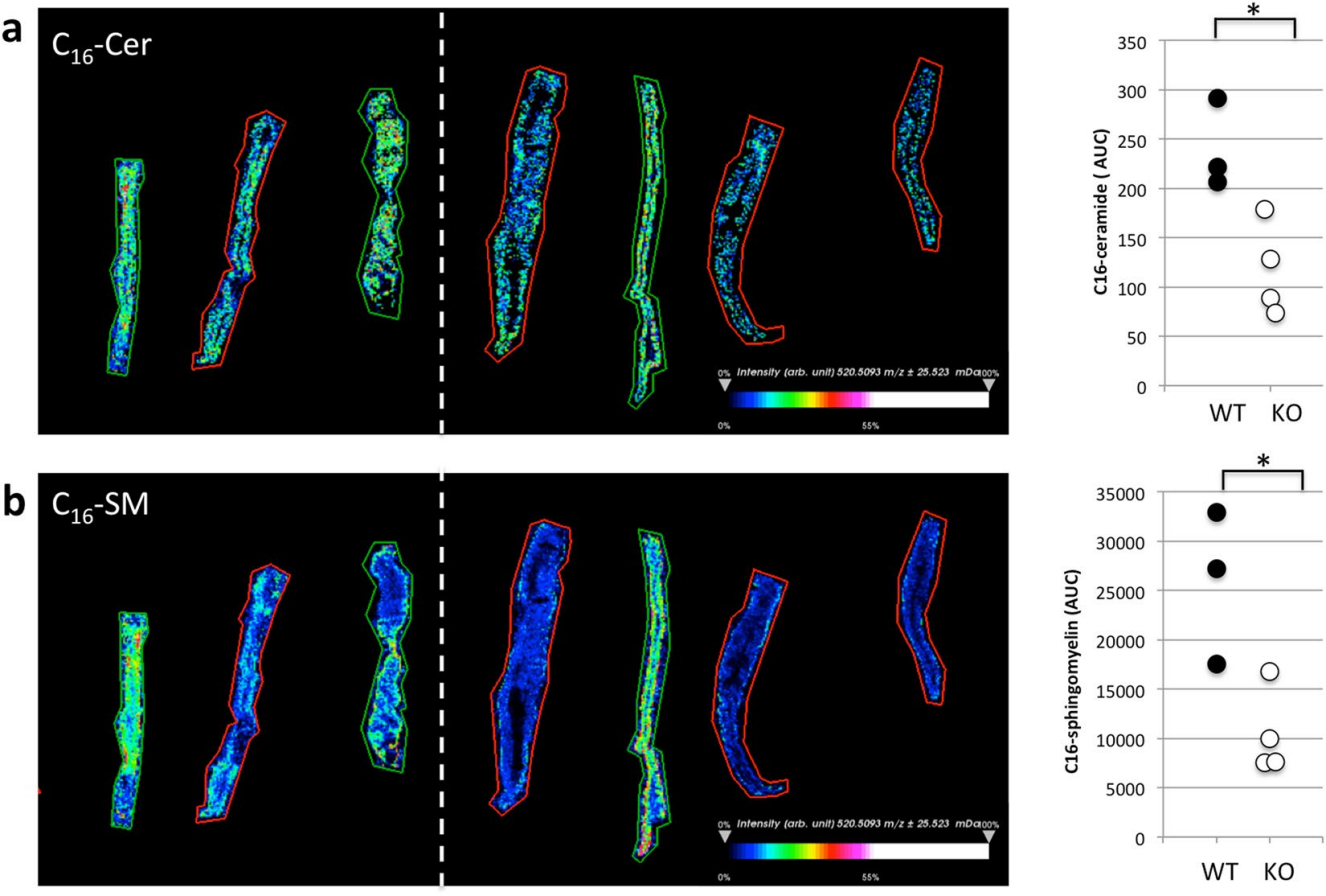
MALDI-IMS of colon tissue following induction of DSS colitis. Colon tissue of DSS treated mice was analyzed by MALDI-IMS and signal (area under the curve, AUC) analyzed for C_16_-ceramide (**a**) and (**b**) C_16_-sphingomyelin. Tissue from wild type and CerS6-deficient mice are indicated by green and red outlines, respectively. This experiment was performed with two sets of female littermates. The white dashed line separates the two sets. *p < 0.05

Next, we analyzed sphingolipids in DSS treated mice. MALDI imaging mass spectrometry (IMS) is a powerful tool to analyze the distribution of proteins and other molecules including sphingolipids in tissue sections *in situ*^16,17^. CerS6-deficient mice have significantly reduced C_16_-sphingolipids^11,15^. This reduced level of C_16_-sphingolipids was also observed in mice exposed to DSS (Fig. 2). Sphingosine-1-phosphate (S1P), a pro-inflammatory sphingolipid that has been associated with colitis^2^, was below limits of detection by MALDI IMS probing. Analysis of serum and tissues by LC-MS showed a similar decrease in C_16_-ceramide (Table 1). Other ceramide species were not significantly altered. Sphingosine did not significantly differ either in circulation or in colon tissue. In untreated wild type or CerS6-deficient mice, serum sphingosine levels are about 25 ng/ml. On day 8 of the DSS protocol, serum sphingosine in wild type mice was 32 ± 8 ng/ml and in CerS6-deficient mice it was 25 ± 7 ng/ml (n = 5). Serum levels of S1P were comparable between wild type and CerS6-deficient mice but a significant increase in S1P was detected in colon tissues of CerS6-deficient mice (Table 1).

**Table 1:** Sphingolipids in DSS treated wild type and CerS6-deficient mice.

	Serum pmol/100 μl		Colon tissue (pmol/mg protein)*1000	
	WT	KO	WT	KO
C14	0.7 ± 0.1	0.5 ± 0.1	35.0 ± 12.8	18.0 ± 5.4*
C16	7.6 ± 1.1	1.7 ± 0.7*	1938.6 ± 552.8	1280.1 ± 145.1*
dhC16	0.9 ± 0.3	0.2 ± 0.0*	146.1 ± 37.6	72.2 ± 21.3*
C18	9.6 ± 3.6	9.4 ± 1.8	258.9 ± 102.6	338.7 ± 83.4
C18:1	0.7 ± 0.5	0.9 ± 0.3	267.7 ± 65.2	300.9 ± 58.4
C20	8.2 ± 0.0	10.0 ± 3.2	164.2 ± 54.8	237.4 ± 40.2
C20:1	1.1 ± 0.4	1.2 ± 0.3	132.4 ± 42.1	130.2 ± 21.3
C20:4	0.0 ± 0.0	0.0 ± 0.0	1.4 ± 0.3	2.0 ± 0.7
C22	11.7 ± 1.0	15.1 ± 2.7	101.4 ± 17.2	136.1 ± 30.8
C22:1	1.4 ± 0.1	1.5 ± 0.1	54.2 ± 14.2	59.9 ± 15.2
C24	15.6 ± 3.9	22.7 ± 5.8	192.1 ± 46.6	182.4 ± 79.2
C24:1	20.8 ± 1.4	16.6 ± 3.5	445.2 ± 54.7	432.4 ± 100.1
C26	0.9 ± 0.2	1.2 ± 0.4	24.7 ± 7.1	17.3 ± 10.6
C26:1	0.7 ± 0.1	0.9 ± 0.2	9.5 ± 3.6	15.1 ± 4.5
Sphingosine	3.2 ± 0.8	2.5 ± 0.7	55.0 ± 12.3	66.3 ± 19.0
S1P	79.0 ± 0.8	81.8 ± 9.0	0.2 ± 0.1	0.5 ± 0.2*

### Neutrophil infiltration is increased in CerS6-deficient mice

**Figure 3:**
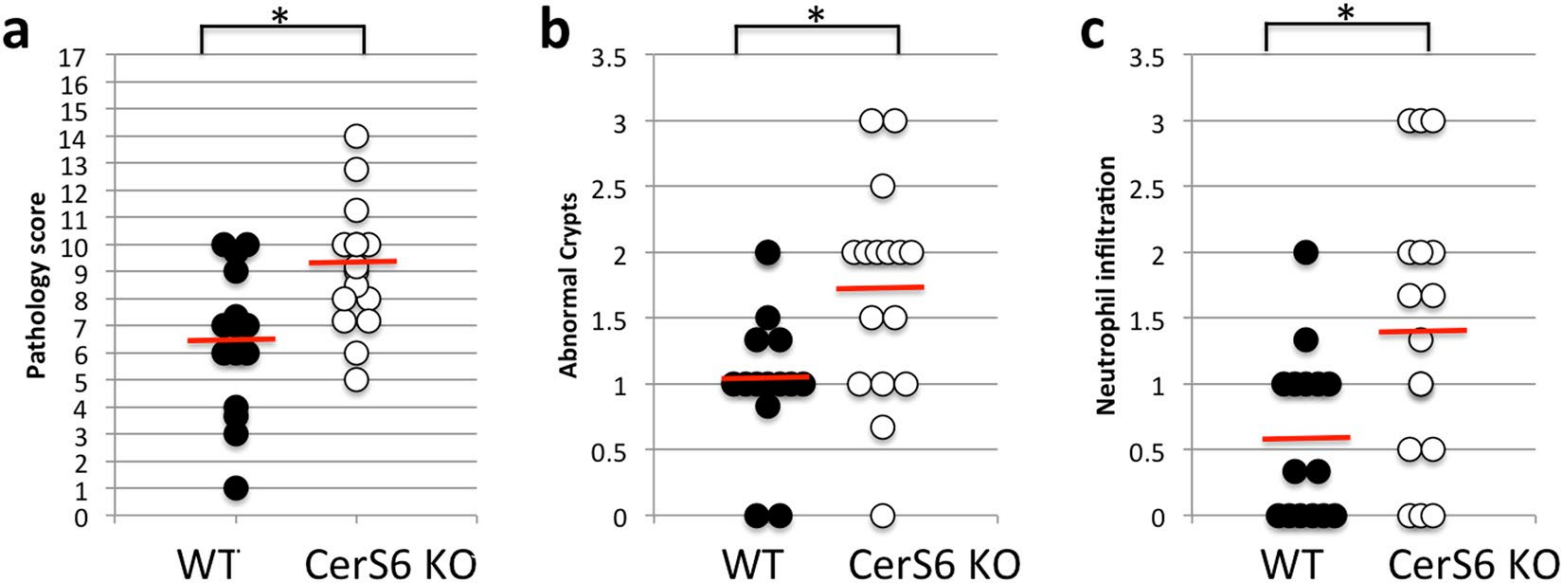
Histological analysis of the colon. (**a**) Total pathology score, (**b**) neutrophil infiltration, (**c**) abnormal crypts. The maximal total pathology score is 17. Neutrophil infiltration and abnormal crypts are sub-score categories of the total score. Data is from 3 independently performed experiments, each with at least n = 4 per group. *p < 0.05

**Figure 4:**
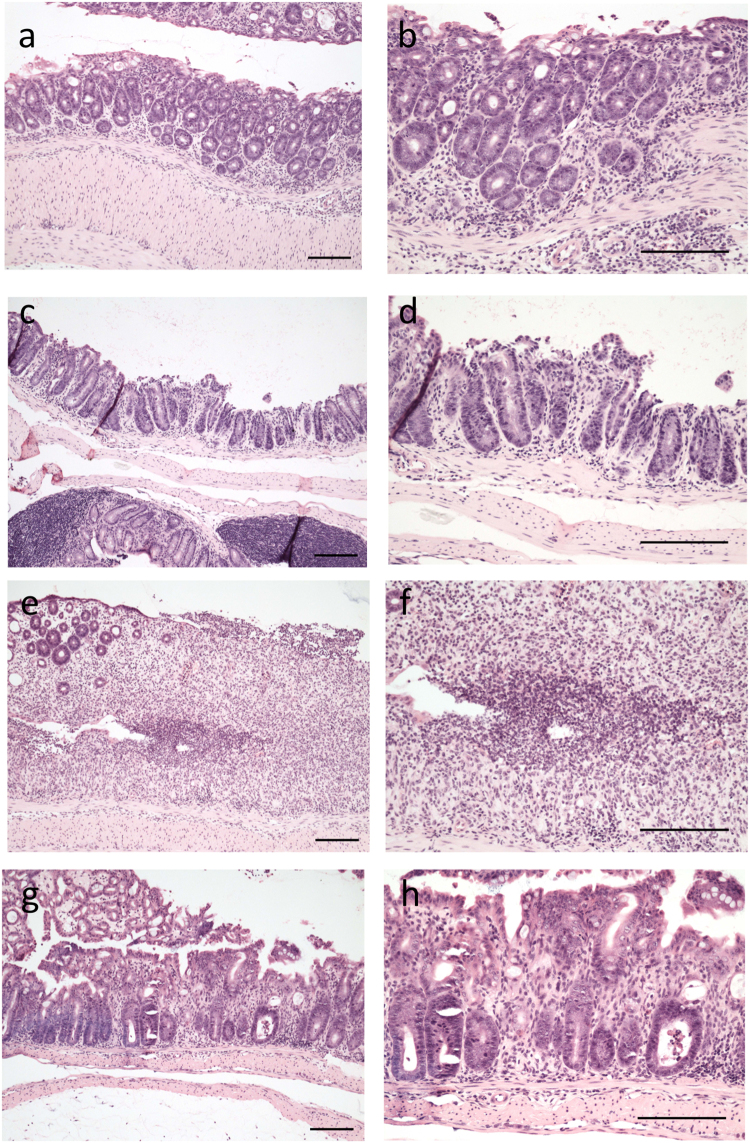
H&E stained colon tissue from DSS treated mice. Images of colon pathology from wild type (**a**–**d**) and CerS6-deficient mice (**e**–**h**) on day 8 of the experiment. Left panels are 10× and right panels are 20× magnification of the same tissue section. Bars indicate 100 μm. Neutrophils in wild type mice are most commonly found adjacent to ulcerated areas (**a**,**b**). Crypt disruption, some ectasia of crypts and some hyperplasia are evident in wild type mice (**c**,**d**). Lamina propria of CerS6-deficient mice contain numerous mononuclear cells and neutrophils with most neutrophils are seen in areas of epithelial disruption (**e**,**f**). CerS6-deficient mice have evidence of crypt disruption, ectasia with crypt abscesses, and numerous mononuclear cells in the lamina propria (**g**,**h**).

Analysis of colon tissue from DSS treated wild type and CerS6-deficient mice indicated that lack of CerS6 was associated with an increased overall pathology score (Fig. 3a). The increased score was primarily driven by a significant increase in abnormal crypts and neutrophil infiltration (Fig. 3b,c). Histopathology of wild type and CerS6-deficient mice is shown in Fig. 4. CerS6-deficient mice exhibited crypt disruption and ectasia with crypt abscesses. Crypt disruption was also observed in wild type mice but to a lesser degree and with only some ectasia and hyperplasia. The lamina propria of CerS6-deficient mice contained numerous mononuclear cells and neutrophils. As in CerS6-deficient mice, neutrophils in wild type animals were most commonly adjacent to ulcerated areas. However, there are fewer areas of ulceration and epithelial disruption in wild type compared to CerS6-deficient mice.

**Figure 5:**
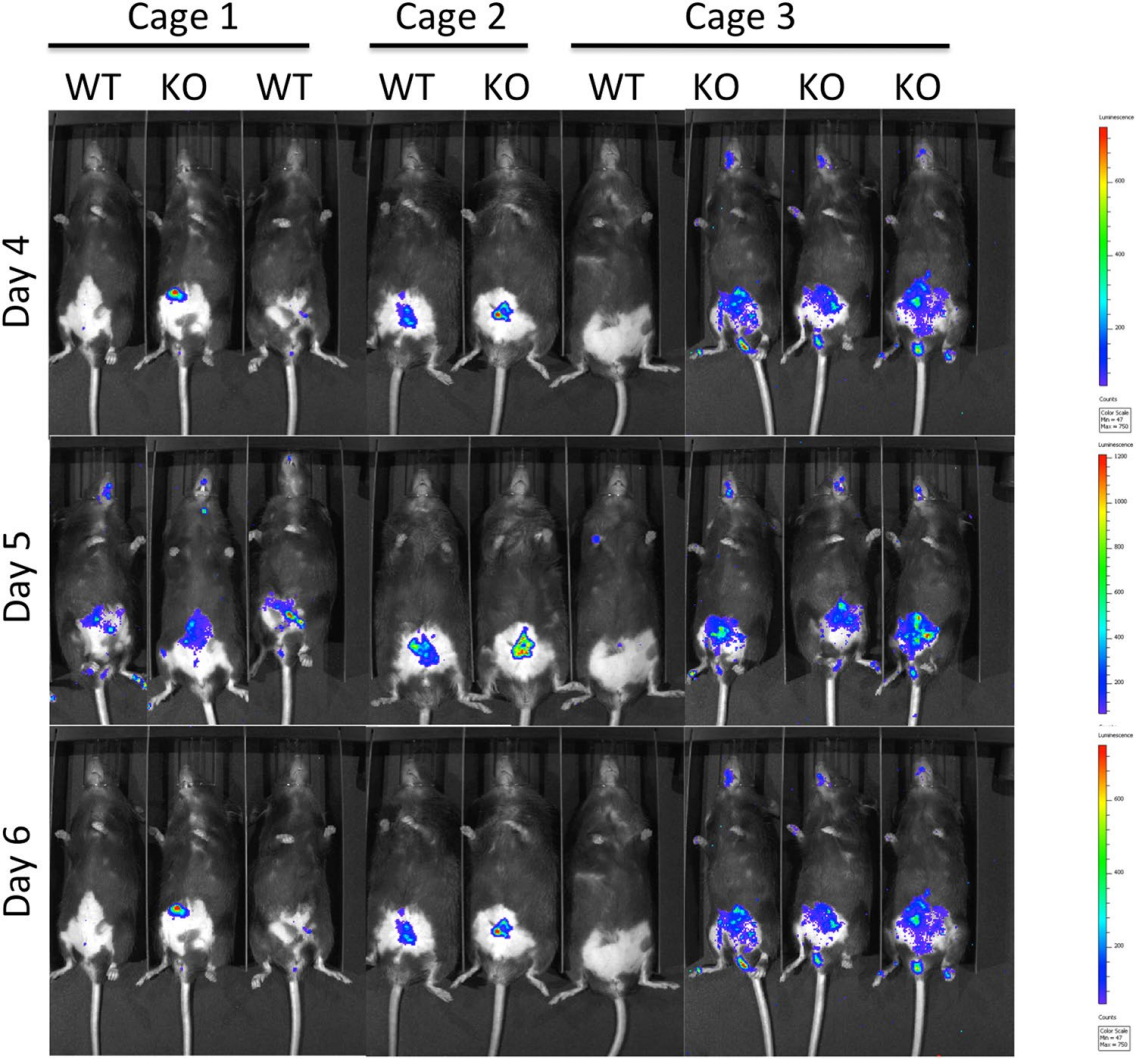
Bioluminescent imaging of myeloperoxidase activity. A subset of mice was injected with a chemiluminescent substrate that detects MPO activity. Mice were imaged 10 minutes post i.p. injection using a 5 minutes exposure time on days 4, 5, and 6. Cages 1 and 3 were female mice. Cage 2 were male mice. Statistical analysis was performed on photons/second from each image.

To confirm differences in neutrophil infiltration, a subset of mice was analyzed for myeloperoxidase (MPO) activity. Mice were injected with a chemiluminescent *in vivo* reagent designed to monitor inflammation by detecting myeloperoxidase (MPO) activity of activated phagocytes. Imaging indicated that MPO activity was limited to the intraperitoneal cavity (Fig. 5). MPO activity in CerS6-deficient mice was estimated to be 2.5 times higher than in wild type mice (95% CI: 1.19, 5.28; p = 0.0036).

### Intestinal permeability in CerS6-deficient mice is similar to wild type mice

**Figure 6:**
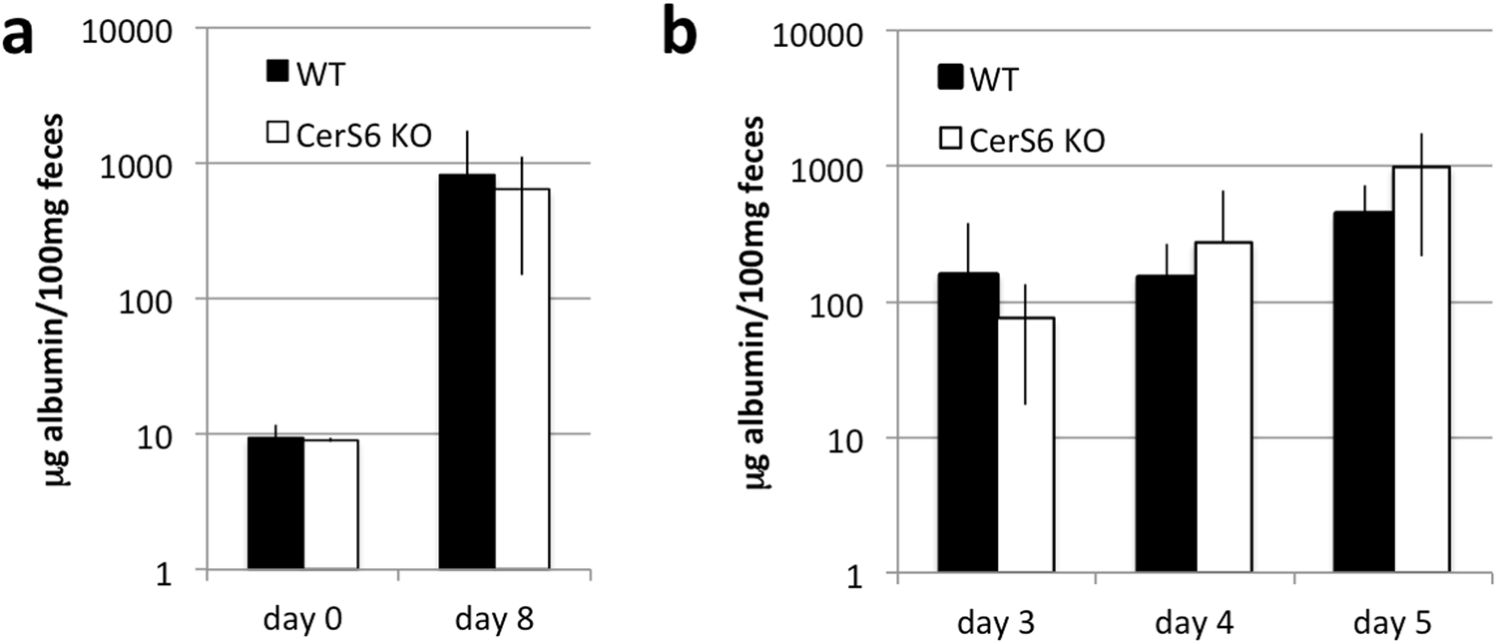
Gut permeability as a measure of fecal albumin. Mouse fecal albumin was quantified by ELISA in untreated mice (n = 2–3) or at the conclusion of the experiment (n = 5) (**a**) and on day 3–5 of DSS induced colitis (3 independently performed experiments each with at least n = 4 per group) (**b**). Data shown are the average ± standard deviation. Differences between wild type and CerS6-deficient mice were not significant.

Pathology and the MPO functional assay suggested that DSS-induced colitis significantly increased the presence of neutrophils in the intestines of CerS6-deficient mice. We therefore investigated the possibility that CerS6-deficient mice have an overall increase in intestinal permeability. Intestinal permeability was assessed by quantifying fecal albumin^18,19^. Fecal albumin levels did not differ between wild type and CerS6-deficient mice prior to DSS treatment (Fig. 6a). In DSS treated mice, levels of albumin increased substantially but to similar extent in both groups of mice at day 8 (Fig. 6a). Evaluation of fecal albumin between days 3–5 during which disease activity scores are higher in CerS6-deficient mice also failed to reveal any significant differences in intestinal permeability (Fig. 6b).

## Discussion

In this study we analyzed the effect of CerS6-deficiency on susceptibility to DSS-induced colitis. Analysis of weight loss suggested there is no difference between wild type and CerS6-deficient mice (Fig. 1a), yet the kinetics of disease activity score indicated a more rapid progression of colitis in CerS6-deficient mice (Fig. 1b). This result was unexpected, since CerS6-deficient mice are protected from development of colitis in the adoptive transfer model^12,15^. C57BL/6 mice are highly susceptible to DSS colitis, thus weight loss may occur at the maximal rate masking differences in disease progression^20^. Future experiments using a mild induction of colitis with 1% DSS may reveal differences in weight loss and could also serve as a model to study the role of T cells during the recovery phase^21^. This is especially relevant as the DSS model, which is classically used as a model of colitis, has recently been recognized as a model that more accurately represents tissue injury.

DSS treated CerS6-deficient mice had reduced levels of C_16_-sphingolipids and the pro-inflammatory sphingolipid S1P was elevated within colon tissue but not in serum (Table 1). A decrease in C_16_-sphingolipids is expected in CerS6-deficient mice, although an increase in S1P has not previously been observed at baseline in liver, lung or kidneys^15^. We therefore hypothesize that increased S1P in colon tissue of DSS treated CerS6-deficient mice is associated with colitis related inflammation. However, future experiments will be required to compare S1P within colon tissue of both untreated and DSS treated mice over the course of disease development.

CerS2 and CerS6 have previously been observed to have opposing effects with regard to apoptotic susceptibility^22^. Similar opposing effects were observed in the EAE model, in which CerS2 null mice were protected while CerS6 null mice had exacerbated disease^14,23^. Different phenotypes in the same model system can be expected, since CerS2-deficient mice, which lack the ability to generate very long chain ceramides such as C_24_-ceramide, have a compensatory increase in C_16_-ceramide while CerS6-deficient mice have reduced C_16_-ceramide^11,24^. Thus it is somewhat surprising that both CerS2- and CerS6-deficient mice are more susceptible to DSS-induced colitis^25^. However, the mechanisms that lead to increased colitis appear to be quite distinct. In CerS6-deficient mice, circulating levels of sphingosine or S1P were comparable at both baseline or following DSS treatment. In contrast, circulating levels of sphingosine and S1P are significantly increased even prior to DSS treatment in CerS2-deficient mice^25^. Oertel *et al*. showed that CerS2-deficiency decreases expression of tight junction proteins, which increases gut permeability^25^. This increase in intestinal permeability leads to elevated immune activation even in the absence of DSS treatment^25^. In CerS6-deficient mice, we did not detect a difference in intestinal permeability prior to DSS exposure and although fecal albumin levels increased with exposure to DSS there was no significant difference between wild type and CerS6-deficient mice (Fig. 6). Thus increased gut permeability could not account for the more rapid increase in disease activity score in CerS6-deficient mice prior to day 5.

Chemical disruption of the gut mucosa with DSS induces inflammation in the absence of B or T cells, although T cells have been shown to play a role in the recovery phase following induction of mild colitis^26,27^. Administration of 3% DSS results in severe colitis with acute inflammation of the colon involving infiltration of granulocytes and macrophages in the mucosa^28^. Macrophages may have played a more prominent role in one wild type animal, which had an average pathology score but no evidence of neutrophil infiltration. In general however, the early influx of neutrophils in the colon has been shown to correlate with tissue destruction on day 8^21^. Histological analysis of colons on day 8 in our study indicated that CerS6-deficient mice had an overall higher pathology score associated with significantly more abnormal crypts and enhanced neutrophil infiltration (Figs 3 and 4). Significantly increased neutrophil MPO activity in CerS6-deficient mice was detected as early as day 4 (Fig. 5). The underlying reason for the enhanced colitis and/or neutrophil infiltration in CerS6-deficient mice will require further analysis. *In vitro* experiments with CerS6-deficient neutrophils have shown that stimulation with IFNγ/TNFα increases nitric oxide, CD11b expression, and adhesion while stimulation with G-CSF increases migration through upregulation of CXCR2 expression^11,14^. The increase in CD11b and CXCR2 expression in CerS6-deficient neutrophils was also observed in an *in vivo* model of multiple sclerosis (EAE model)^14^. In this model, CerS6-deficient mice experienced exacerbated disease that was associated with increased neutrophil infiltration into the central nervous system^14^. Cytokines and chemokines (CCL2, CCL5, CXCL2, IL-17, Il-6) as well as iNOS were also significantly increased upon EAE induction in CerS6-deficient mice^14^. We speculate that neutrophils in DSS treated CerS6-deficient mice may have a similar increase in CD11b and CXCR2, which would be indicative of enhanced activation and migration, and offer a potential explanation for the more rapid onset of disease activity during days 1 and 5 (Fig. 1b).

In conclusion, we have shown that CerS6-deficient mice are more susceptible to DSS-induced colitis, which contrasts with the protective effect that lack of CerS6 has in adoptive transfer models of colitis. Studies with CerS6-deficient mice suggest that this protein has different functions in distinct immune cell subsets, which will complicate efforts to develop specific inhibitors. Generation of tissue-specific CerS knockout mice is expected to further elucidate the roles of CerS family members.

## Methods

### Animals and DSS induced colitis

All animal experiments were performed with approval by the Institutional Animal Care and Use Committee at the Medical University of South Carolina to ensure that ethical regulatory and policy mandates governing the use of animals in research are met (Animal Welfare Assurance #A3428-01). All methods were performed in accordance with the relevant guidelines and regulations. CerS6-deficient mice (C57BL6) were generated by the Texas Institute of Genomic Medicine and obtained from Dr. Besim Ogretmen. Mice were maintained through heterozygous breeding. Genomic tail DNA was used to genotype offspring by PCR. Primers were as follows: 5′ TTCGGTTAAGAATGGCCTTG3′; 5′CACACCCATATGGAACTCTTACA-3′; and 5′-CCAATAAACCCTCTTGCAGTTGC-3′. Expected PCR products are 460 bp for wild type, 295 bp for CerS6 knockout, or both for heterozygous animals. The C57BL/6 strain is highly susceptible to DSS colitis with no significant differences in the frequency of ulceration between sexes^20^. To induce colitis, 3% DSS w/v (MP Biomedicals, cat no. 160110) was added to the drinking water for 5 days with a water change on day 3. Mice were returned to regular drinking water on day 5. During the experiment weight was monitored daily and feces collected. The percentage of weight loss was calculated by dividing body weight on the specified day in accordance with body weight on day 0 (baseline). Disease activity scores were assigned as follows: 0 = normal stool; 1 = soft stool; 2 = very soft stool with blood; 2.5 = watery stool but no rectal bleeding; 3 = watery stool with rectal bleeding. On day 8 mice were humanely euthanized and colon tissue prepared for histological analysis, LC-MS or MALDI-IMS. Serum was collected for LC-MS.

### Bioluminescent Imaging

*In vivo* imaging studies fur was removed with depilatory cream for better visualization of the signal. The XenoLight RediJect Inflammation Probe (Perkin-Elmer, cat no. 760536) was injected intraperitoneal (i.p.) at a concentration of 200 mg/kg. Mice were imaged using the IVIS200 system at 10 minutes post i.p. injection using an exposure time of 5 minutes. MPO activity (photons/second) was quantified using Living Image software.

### Histology analysis

Colons were removed, cleaned, and fixed in formalin. Sections were placed in cassettes and cut longitudinally for histological analysis. Tissues were analyzed for lamina propria inflammation (0–3), goblet cell loss (0–2), abnormal crypts (0–3) and crypt abscesses (0–1), mucosal erosion and ulceration (0–1), submucosal spread to transmural involvement (0–3) and the number of neutrophils counted at x40 magnification (0–4) for a maximal score of 17^29^.

### Sphingolipid analysis by LC-MS and MALDI-IMS

Ceramide species (C_14_, C_16_, C_18_, C_18:1_, C_20_, C_20:1_, C_20:4_, C_22_, C_22:1_, C_24_, C_24:1_, C_26_ and C_26:1_ were quantified by LC-MS as previously described^30^. For MALDI-IMS, colons were collected, feces removed, and tissue wrapped into aluminum foil and flash frozen in liquid nitrogen vapor and stored at −80 °C until analysis of lipids by the MUSC Proteomics Center. Tissues were processed by embedding them into chilled 2% carboxymethylcellulose and flash frozen using dry ice powder. Following overnight storage at −80 °C, cryosections were cut at 10 µm and thaw mounted onto ethanol-cleaned indium tin oxide coated microscope slides. Tissues on slides were coated with a chemical matrix (25 mg/mL 2, 5-dihydroxybenzoic acid with 3 mM KCl and 70% methanol in 0.1% trifluoroacetic acid). Matrix was sprayed onto tissue using an automated sprayer (TM-Sprayer, HTXImaging) with 10 passes at 60 °C, 3 mm offset, 1200 velocity, 10 psi and a 40 mm distance nozzle tip to slide surface. Data were acquired in positive ion mode using a Fourier Transform Ion Cyclotron Resonance (FT-ICR) mass spectrometer (7 Tesla solariX, Bruker Daltonics) equipped with a matrix-assisted laser desorption ionization (MALDI) source. The SmartBeam II laser settings used a total of 500 shots per pixel operated using the smartwalk feature set to 25 µm with one scan per pixel. Stepsize between sampling was 100 µm. Time domain was set to 1 M word with a mass range of 200–1,600 m/z, resulting in a 1.2059 transient with a calculated resolving power of 160,000 at m/z 400. Ion accumulation time was 0.1 second. Data were processed and visualized using SCiLS Lab Imaging Software (Bruker Daltonics, version 2017a. Databases of lipid classes were made from LipidMaps by searching “ceramides” (42 species), “sphingomyelins” (19 species), lyso-glycerophosphocholines (6 species), and lyso-glycerophosphoethanolamines (27 species). Accurate masses of lipids with adducts (M + H, M + Na, M + K, M-H2O + H) were calculated using the software IsotopePattern (Bruker Daltonics).

### Albumin ELISA

Feces were dissolved in sample dilution buffer (50 mM Tris, 0.14 M NaCl, 0.05% Tween20, pH 8.0) to a concentration of 100 mg/ml. Albumin levels were quantified using the mouse albumin ELISA kit (Bethyl Labs) according manufacturer instructions and results quantified using a FluoStar Optima plate reader.

### Statistical Analysis

Two-sample t-tests were used to compare means between wild type and CerS6-deficient groups. Statistical significance was defined by a two-sided alpha level of 0.05. Disease activity score was analyzed with mixed effects linear regression to account for repeated measures over time. A model was fit, which allowed for each group to have a slope between day 1 and day 5, and another slope between day 5 and day 8 (i.e. a spline model with a knot at 5 days and interactions between time effect and group (WT vs. CerS6 KO). Wald tests were used to compare slopes between groups for each time period. For analysis of bioluminescent imaging, a 3-factor repeated measures Anova was used to evaluate difference between inflammation in wild type and CerS6-deficient mice, accounting for trends over time and cage effects. Due to skewness, a log transform of MPO activity was used as the outcome variable.
